# Editorial Note: Cross-Serotype Immunity Induced by Immunization with a Conserved Rhinovirus Capsid Protein

**DOI:** 10.1371/journal.ppat.1012213

**Published:** 2024-05-06

**Authors:** The PLOS Pathogens Editors

After this article [1] was published, concerns were raised about results presented in Figures 1 and S1.

Specifically:

- The ladder panel in Figure 1A appears similar to the ladder panels in Figs. 7A, 8A, and 9 of a *Cellular and Molecular Life Sciences* article [2] by a different research group.
- In Figure S1E, when levels are adjusted to visualize the background, there appear to be discontinuities in the background between lanes 1 and 2, and at the bottom of lanes 2–4.

In addition, there appears to be an error in the labeling of the protein marker in Fig 1A; the methods section states that the molecular weight standard SeeBluePlus2, Invitrogen, was used. The product information for this marker [3] suggests that the top lane should be labeled as 198kDa instead of 188kDa.

PLOS followed up with the authors about these concerns. Regarding the concerns with the ladder in Figure 1A, the corresponding author stated that the ladder is a reproduction of an image provided by the ladder supplier (Invitrogen) to illustrate the bands. Furthermore, the corresponding author stated that the product information in relation to the top marker of the ladder may have changed since the publication of [1].

Regarding the concerns with Figure S1E, the corresponding author confirmed that the Figure S1E panel was prepared using spliced data and provided the original underlying image data, available in the S1 File provided with this notice. The original image data show that non-pertinent lanes were spliced from the published panel but confirm the reliability of the published results.

The corresponding author did not comment on the availability of the remaining data underlying the published results.

The *PLOS Pathogens* Editors issue this Editorial Note to inform readers of the above issues, which the journal considers resolved with the provision of the S1 File available with this notice.

## Supporting information

S1 File Original image data underlying Figure S1E. (PPTX)
