# Supplementary material for: Editorial Note: Cross-Serotype Immunity Induced by Immunization with a Conserved Rhinovirus Capsid Protein
Source: PLoS Pathog. 2024 May 6;20(5):e1012213. doi: 10.1371/journal.ppat.1012213 (PMC11073816; doi:10.1371/journal.ppat.1012213)
Supplement: S1 File — (PPTX) [file ppat.1012213.s001.pptx]

## Slide 1
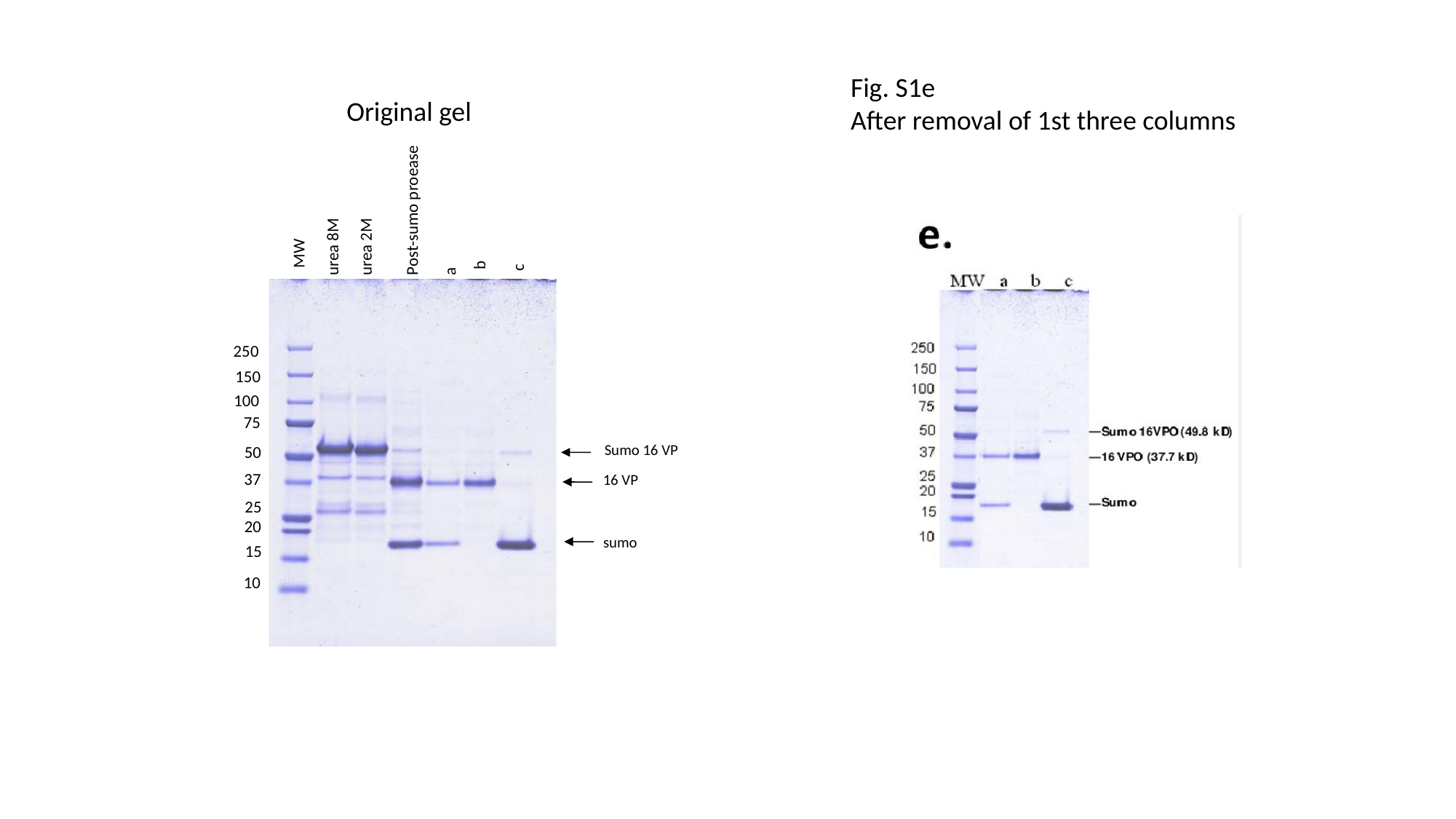

Fig. S1e
After removal of 1st three columns
Original gel
c
Post-sumo proease
a
urea 8M
urea 2M
b
250
150
100
75
50
37
25
20
15
10
MW
Sumo 16 VP
16 VP
sumo
